# A Quiet Hero, An Epic History: A Review of *Tore Godal and the Evolution of Global Health* and a Conversation with Economist Dean Jamison

**DOI:** 10.4269/ajtmh.23-0853

**Published:** 2023-12-26

**Authors:** Claire Panosian Dunavan

How many people know the kaleidoscopic career of Tore Godal--a visionary scientist and medical statesman who, for decades, was a key figure at the intersection of tropical disease research, capacity-building, and policy, financing, and implementation? For many who have witnessed global health’s epic shifts of the last 40 years, Godal needs no introduction.

For others, Conrad Keating’s new book—*Tore Godal and the Evolution of Global Health*—could prove a startling revelation. Using Godal’s life and work as a through-line, Keating narrates six transformative initiatives which might never have launched or achieved their truly remarkable results were it not for the tireless efforts of this quiet, driven Norwegian.

Here are the initiatives in chronologic order:

**The Special Programme for Training and Research in Tropical Diseases (TDR).** In 1974, the World Health Organization (WHO), the World Bank, and the UN Development Program (UNDP) created TDR as an independent unit meant to reduce infectious diseases by strengthening capacity and translating research in disease-affected countries into meaningful interventions. Starting in 1976, TDR was led by Adetokunbo Lucas; a decade later, Godal succeeded Lucas as TDR’s Director and ran the program until 1998. Over both men’s tenure, TDR targeted leprosy, malaria, onchocerciasis, lymphatic filariasis, African trypanosomiasis, Chagas disease, leishmaniasis, and schistosomiasis, among others. But with Godal at the helm, TDR also grew in new, creative ways.

**The African Programme for Onchocerciasis Control (APOC).** Between 1987 and 1989, community trials in onchocerciasis-endemic countries dispensed 120,000 doses of ivermectin (Mectizan), a breakthrough treatment for onchocerciasis; under Godal, TDR individually funded studies in Liberia, Cameroon, Malawi, Guatemala, and Nigeria. At this time, however, WHO had not yet embraced public-private partnerships (PPPs). It was not until TDR forged a historic collaboration that WHO began, in 1995, to work with 31 Ministries of Health and NGOs to coordinate annual mass treatment in 16 sub-Saharan African countries using ivermectin donated by Merck . The Mectizan Donation Program – a coalition that did not answer to WHO, UNICEF or any other UN agency—is now thought to have protected as many as 100 million people from onchocerciasis. As TDR’s Director, Godal also promoted local ownership and engagement through community-based distribution of ivermectin; twenty years later, this pioneering approach had produced 150,000 newly-trained African health workers.

**Roll Back Malaria (RBM).** In 1998, Godal left TDR to become the first director of RBM, a global initiative launched by WHO, the World Bank, UNICEF and UNDP; RBM was also backed by development agencies, banks, private sector groups, and researchers. Although RBM’s initial target (to halve the worldwide malaria toll by 2010) was not reached until roughly 2015, it achieved many impressive goals: the accelerated diagnosis and treatment of malaria with new, effective drugs; more robust malaria prevention, especially through the use of insecticide-treated bednets; and health-sector strengthening, civic involvement, and research around other tools and implementation.

**The Global Alliance for Vaccines and Immunisation (GAVI,** now **Gavi—The Vaccine Initiative).** Officially inaugurated on 31 January 2000 at the World Economic Forum in Davos, Switzerland, GAVI was the first international organization to connect the public and private sectors around the shared goal of ensuring that essential vaccines reach future generations of vulnerable children. Godal worked directly with the newly-created Bill and Melinda Gates Foundation (BMGF) in order to secure the program’s initial five-year, 750 million dollar investment. He subsequently served as GAVI’s director until 2006. By 2019, when GAVI received the Lasker-Bloomberg Public Service Award with Tore Godal and former WHO Director General Gro Harlem Brundtland in attendance, GAVI had aided in vaccinating more than 760 million children and saved an estimated 13 million lives in 73 countries. Today, GAVI protects health and saves lives by increasing the equitable and sustainable vaccination of people of all ages.

**Maternal and Child Health (MCH).** In 2005, Tore Godal began to focus on lowering maternal mortality. After first arranging for Norway to partner with India in a project that incentivized Indian women to give birth in clinics and hospitals, Godal helped promote a new results-based “ecosystem” in which prominent political leaders aligned with specific UN Millennium Development Goals (MDGs). In the United Kingdom, Gordon Brown prominently launched a global “compact” in support of the Health Millennial Goals while Barack Obama and Angela Merkel, among others, threw particular weight behind a “Women and Children First” agenda; the latter also drew strong support from UN General Secretary Ban-ki Moon, BMGF, and global media stars. Between 1990 (the baseline year for MDGs) and 2015, child and maternal deaths fell by 50%.

**The Coalition for Epidemic Preparedness Innovations (CEPI).** It was 2013’s dramatic epidemics of Ebola virus in West Africa that motivated Godal and others to found CEPI, an organization intentionally housed in Norway as opposed to Geneva. When CEPI officially launched in 2016, it received start-up funding from GLOBVAC, a fund created ten years earlier by Norwegian Prime Minister Jens Stoltenberg to support Norwegian-led research on vaccine development and deployment. CEPI subsequently received funding from public, private, philanthropic, and civil society organizations in order to finance many independent projects aimed at developing vaccines against emerging infectious diseases.

Now for some backstory. No one would dispute that many talented, visionary people from diverse countries, continents, and sectors—public health, medicine, economics, pharma, and philanthropy—were seeking innovative ways to save lives and reduce illness among the world’s poorest people well before Godal helped launch the programs described above. But huge hurdles remained before specific agendas took shape and the programs got financed and began to achieve results.

Enter a physician and immunologist born just before World War II. Soon after, his native land was occupied by the Nazis, leading to hardships and darkness. While growing up in Norway, Tore Godal’s earliest dream was to work as a district medical officer in the rural valley of his birth. Decades later, to quote Keating, the same Norwegian doctor became “the closest thing we have to the World’s District Medical Officer.” In a similar vein, in 2019, The Lancet profiled Godal and christened him a “quiet colossus of global health.”

In short, it was that quiet but extraordinarily effective leader and public servant who transformed what could have been pipe-dreams into global realities and was also determined--again quoting Keating--to “translate ideals and ideas into constructive actions, therapeutics and drugs on the ground …” thus tangibly improving the lives of people in poor, remote settings.

But first came rigorous preparation, starting with medical and doctoral degrees from the University of Oslo, followed by training and mentorship by Morten Harboe, Norway’s leading immunologist. In 1970, Godal moved to Ethiopia to conduct leprosy research at the newly-opened Armauer Hansen Research Institute. From the mid-1970s to the mid-1980s, he chaired WHO’s steering groups for immunology research in leprosy and tuberculosis, followed in 1986 by his appointment as Director-General of TDR. Then came Part Two of Godal’s global career, in which he performed further high-level functions in Geneva while, at different times, working in the Office of Norway’s Prime Minister and Ministry of Foreign Affairs and as a special advisor to WHO Director General Gro Harlem Brundtland. In 1999, following his mandatory retirement from WHO, Godal helped launch GAVI (and subsequently chaired it for another five years). That same year, he was a co-recipient of the Prince Mahidol Award in Public Health and in 2019 received the King of Norway’s Medal of Merit.

So how does a medical historian tell the story of a man of science and healing who—both publicly and behind closed doors—brokered unprecedented international partnerships and oversaw their financing and implementation while remaining deeply connected to on-the-ground stakeholders?

As in several of his previous books^1^, the author of *Tore Godal and the Evolution of Global Health* accomplishes this with extensive reporting and eye-witness details, memories, and quotes which yield a rich, human portrait as well as some unique challenges Godal faced both in boardrooms and “end of the road” settings where people’s lives literally hung in the balance. As a result, the 200-page book Keating recently added to the Routledge Series in the History of Science, Technology and Medicine is a richly-layered narrative of modern global health history which deserves to be read by many people, both in academia and beyond. In short, not only does its engaging, well-paced text depict an insightful scientist who was both principled and politically astute, it also summarizes 40 years of policy and progress and, in doing so, presents important lessons for future generations.

*Tore Godal and the Evolution of Global Health* ends with 2016’s successful deployment of an Ebola vaccine whose efficacy was confirmed in a CEPI-organized trial led by WHO, the Guinean Health Ministry, and Norway’s Institute of Public Health. Today, according to a UNICEF website^2^, WHO’s Emergency Vaccine Stockpile contains roughly 500,000 doses of the Ervebo Ebola vaccine manufactured by Merck. Since 2016, CEPI has continued to play a pivotal role in the accelerated development of vaccines against pandemic diseases including COVID-19.

A final layer to Keating’s story? Before describing the highly-impactful initiatives Godal helped to shape, Keating outlines other factors that likely contributed to his resolute-yet-selfless nature, best described as far more focused on “getting the right things done than emphasizing who did them.”

First there’s Norway itself: a nation, Keating reminds us, whose history has never been tainted by possessing overseas colonies. Of course, no country is perfect. But to this day, Norway remains known to many for its traditions of hard work, solid governance, social justice, and global generosity^3^ to people beyond its borders less fortunate than its own.

Then there is Godal’s family. Tore’s father Odd Godal—a Lutheran pastor who bravely sheltered Norwegian saboteurs who planted explosives and partly destroyed Europe’s largest heavy-water plant (thus thwarting Hitler’s early hopes to develop an atomic bomb)—certainly played a role in molding his son’s like-minded instinct “to do radical things if you want to change the world…” Meanwhile, Godal’s maternal line (which included an artistic mother and a grandfather who was a Nobel-nominated novelist and playwright) contributed creativity and wonder.

Godal’s conviction that scientific evidence should guide global health policy was a final guiding star supported by facts and examples in Keating’s book. One among many is a moment in the early 1990s, when, frustrated by WHO’s ambivalence, Godal decided that TDR should entirely underwrite (thus expending nearly all of its field research budget) a 2-year, multi-country study in Africa strategically designed to test whether insecticide-treated bednets would save children’s lives in certain malarious settings. The answer, according both to the original cluster-randomized trials and later analyses, was an unambiguous “yes.”

A long-time colleague of Tore’s is Dean Jamison, whose own career-milestones include his appointment by Lawrence Summers (then chief economist of the World Bank) to serve as the principal author of the Bank’s 1993 World Development Report (WDR93) entitled “Investing in Health.” This publication proved ground-breaking with respect to the Bank’s evolving views on how best to support economic development in low- and middle-income countries. That same report was arguably just as influential in the founding of the Gates Foundation, with whom Godal later collaborated in multiple ventures, starting with GAVI.

Not long ago, I visited with Jamison to discuss his memories of Tore Godal and impressions of Keating’s book. What follows is a lightly-edited transcript of our recent conversations in October and November 2023.

## A FRIEND’S REFLECTIONS ON TORE GODAL

**First of all, let’s go back in time. Dean, when did you first meet Tore and what are some of your earliest memories of him?** My earliest memory of Tore involves Barry Bloom, then-Dean of the Harvard School of Public Health. Barry was chairing the TDR Advisory Committee on which I served and Tore was TDR’s Director. To his credit, Barry was very committed to spending time at WHO even though he was a basic scientist.

From the beginning, what I remember about Tore was how strongly he believed in scientific institutional development as a TDR mandate. At that time, I believe he put about 25 percent of its resources into it. What I later learned from our review process was that the young scientists, say from Mali--after they got their PhDs--often went home to scientifically strong institutions and worked in labs or as epidemiologists.

But some of the time, they wouldn’t continue very long in basic science or medicine, and this led to some criticism of TDR. But as Tore said to me: ‘Dean, don’t you think we want scientists over there in the Ministry of Health or even working in the private sector? Isn’t that a success of our program as much as that person’s research appearing from time to time in the American Journal of Tropical Medicine and Hygiene?’ It sounded right to me then. It still sounds right to me.

**So even then Tore was savvy and pragmatic?** Yes, very pragmatic. Keating has accurately nailed his temperament. It was very pragmatic, mission-oriented and politically savvy while always focused on honorable goals.

In fact, Tore is very Norwegian in not being self-aggrandizing; it strikes me as a national characteristic. This doesn’t mean that Norwegians aren’t ambitious or patriotic. In fact, it’s a country with even more flags all over the place than our own. But the Norwegians also have a view, as did Gro Harlem Brundtland,^4^ that Norway had a role to play in the world and a responsibility and capacity to do it well.

Lastly, Tore was always totally honest. Even when the honesty didn’t work to his advantage (and I saw this a few times), well, the honesty always won. He would tell the story and accept the situation as it was rather than trying to change things around.

**What do you recall about Tore’s early work as Director of the Armauer Hansen Leprosy Research Institute, a chapter which preceded his TDR tenure?** It reminds me that Norway is still a religious country with longstanding missionary roots in Ethiopia. In addition, as I understand it, the leprosy center where Tore worked was engaged in setting up both clinical care and leprosy research, and that Tore himself made major contributions on the clinical side while also advancing the science around the pathogenesis of leprosy.

What I later observed in Tore was that, without wearing it on his sleeve, he had great empathy for disadvantaged people and a continuing passion to help them, particularly through science.

Here’s an interesting connection to leprosy research and Tore’s empathy for leprosy patients that relates to “*Investing in Health Research and Development*,” the 1996 WHO Ad Hoc Committee on Health Research Relating to Future Intervention Options (unpublished document TDR/Gen/96.1). That was a committee created by Tore and James Tulloch^5^ which I chaired. It was interesting to me that Keating points to the committee’s work as having been very influential in Tore’s own thinking.

Well, in that committee, which was really fun and interesting, one of the questions we discussed was the economics of an intervention that had a given set of characteristics and--if you could develop a vaccine with those characteristics--how attractive would it be? One of my conclusions was that a leprosy vaccine would not be very attractive at all, because when you have a low incidence of disease, the vaccines just don’t look very good. To an economist like me, if a disease is not economical to prevent but you have a pretty good chance of curing it, you’re better off waiting until somebody gets it, then treat them. But you don’t say that too much in public.

Anyway, in the aftermath of that work, I did say something to that effect and it was quoted and somehow got into print, and Tore saw it. He quickly got back to me to say that the world absolutely *did* need a leprosy vaccine [Jamison laughs].

**You yourself have had epiphanies around the importance of investing in science as part of any health development agenda. So now let’s hark back even earlier to “Investing in Health,” the World Bank Development Report of 1993.** Well, first of all,18 months after Larry Summers gave me that assignment, there was a new vice president and chief economist at the World Bank named Michael Bruno—a guy totally different than Larry in that he really didn’t care about technical things. Most macro-economists are like that: they think in a more abstract way.

Michael asked me an interesting question, especially since—while interviewing me for the job of leading WDR 93--Larry Summer had explicitly asked what my three main conclusions might be. ‘I’ve read the WDR summary,’ Michael said, ‘and I assume you went into the year of writing having a pretty good idea where you were going to come out, but what I want to know is this. What did you learn in the space of a year that you *didn’t* expect?’

And the answer to that question was really easy. It was about the role of science in the evolution of human health—not social determinants of health, not therefore income or other things like that. Not that they were unimportant, but it was the science that created the diagnostics, the vaccines, the drugs… and the science that underlay how to sensibly message changes in behavior like ‘don’t smoke’ or “pasteurize milk.’

Ironically, the science that continues to drive changes in human health was not where we focused in WDR 93. But I do think the report helped provide the rationale for investing in science, which is what led to my continued contact with Tore.

**Dean, a few years later you got involved in the economics of new treatments for malaria. This brings me to early decisions Godal made around artemisinins. Can you elaborate on that?** If you remember, the original work on artemisinins was done in southeast Asia and Vietnam; in 2015, a Nobel Prize was awarded to Professor Youyou Tu, a Chinese woman scientist who helped identify active parts of the botanical. Looking back, I believe that taking those early findings about the potency of artemisinins and moving them forward into practice was one of the most important things Tore did as TDR Director.

This is very different from the bednet story because, in this case, he went ahead of the clinical trials to the clinical experience that, I think in Vietnam, was very compelling, then concluded that--in places where we were losing chloroquine--artemisinins were a viable alternative.

But I still remember our conversations where he said it needed to be a risk-risk calculation. In other words, what was the risk of using an artemisinin when its efficacy had not yet met generally-accepted medical standards. By those standards, the drugs had not yet progressed far enough for The New England Journal of Medicine to say: ‘this drug is ethical to use.’ But then Tore would say: ‘How ethical is it *not* to use an artemsinin when you have strong and compelling evidence that the lack of effective antimalarials in parts of the world that are losing chloroquine is killing people in large numbers. You need to make that judgment. You need to make that trade-off.’

As a result, he and TDR not only moved artemisinins into trials but got them out into use because the situation was urgent.

**Final question. In summary, what would you like to say about Tore’s example and legacy?** In early October, I had an interesting afternoon with Tore in New York while taking a walk with him through Central Park. He was older of course and speaking more softly than he used to. Nonetheless, even though I’m not exactly answering your question, what first comes to mind is something I would like in my own future… namely, to be as fully and intellectually engaged as he was in our questions and discussion on that day.

I was particularly interested in where we were in COVID vaccines and what we knew about the non-mRNA class, a lot of which have been developed internationally and seem to work pretty well.

One of the things I wanted to learn from him was his sense of the extent to which the mRNA approach—the kind of high technology that’s not that easily replicated in a developing country—was likely to displace or divert attention from the power of existing approaches to vaccine development. I also had related questions around the use of existing live, attenuated vaccines to generate innate immunity while waiting for a pathogen-specific vaccine—for example, against a new strain of COVID or influenza. And how to stay engaged with those questions, which entails grappling with issues involving both immunology and vaccine development.

Tore thought hard and gave me long and thoughtful answers. He was obviously motivated to consider those questions and was still as intellectually engaged as ever at the intersection of science and public policy. But it was mainly the science-weighted part of that intersection. Four years from now, when I’m 84 years old, I hope for myself that I’m still as engaged.

This thought returns me to Conrad Keating’s book, and how profoundly it captures the contributions and personality of Tore Godal. Tore accelerated scientific advances of great consequence for human welfare. Perhaps ironically, his coolly detached honesty and pragmatism created an agenda of health for the poor and disadvantaged far more effective than the rhetoric of universal health care, valuable as that can be. What pleases me most is that Keating’s book will allow a younger generation of scholars and activists to learn from Tore’s example.[Bibr b1][Bibr b2][Bibr b3][Bibr b4][Bibr b5]

## Footnotes

^1^
Conrad Keating was a Writer-in-Residence and Visiting Professor at the School of Medicine, Trinity College Dublin while writing *Tore Godal and the Evolution of Global Health*. He works on the social history of medicine and the history of science and was previously the Writer-in-Residence at the Wellcome Unit for the History of Medicine at the University of Oxford. His most recent publication is *Anthony Cerami: A Life in Translational Medicine* (2021). Previous works include *Kenneth Warren and the Great Neglected Diseases of Mankind Programme: The Transformation of Geographical Medicine in the US and Beyond* (2017); *Great Medical Discoveries: An Oxford Story* (2013); and *Smoking Kills: The Revolutionary Life of Richard Doll* (2009). Keating has an ongoing ten-part *Art of Medicine* essay series on the history of randomized controlled trials in *The Lancet*.

^2^

https://www.unicef.org/supply/media/19646/file/EbolaStockpileAvailabilityReport0611203.pdf

^3^
Since 1976, Norway has met the UN’s target of 0.7% ODA/GNI (overseas development spending/gross national income) and is committed to maintaining spending at around 1% of its GNI, which it has met almost every year since 2013. For more, see https://donortracker.org/donor_profiles/norway#oda-spending

^4^
Brundtland was a medical and public health doctor who became a 3-term Prime Minister of Norway, then served 5 years as Director-General of WHO.

^5^
At the time, Tulloch was the WHO Director of Child Health and Development.
